# Environmental tobacco smoke in hospitality venues in Greece

**DOI:** 10.1186/1471-2458-7-302

**Published:** 2007-10-23

**Authors:** Constantine I Vardavas, Barbara Kondilis, Mark J Travers, Elisabeth Petsetaki, Yiannis Tountas, Anthony G Kafatos

**Affiliations:** 1 Department of Social Medicine, Faculty of Medicine, University of Crete, Greece; 2 Hellenic American University, Athens, Greece; 3 Roswell Park Cancer Institute, USA; 4 National School of Public Health, Athens, Greece; 5 Center for Health Services Research, School of Medicine, University of Athens, Greece

## Abstract

**Background:**

Exposure to environmental tobacco smoke is a major threat to public health. Greece, having the highest smoking prevalence in the European Union is seriously affected by passive smoking. The purpose of this study was to measure environmental tobacco smoke (ETS) exposure in the non smoking areas of hospitality venues and offices in Greece and to compare the levels of exposure to levels in the US, UK and Ireland before and after the implementation of a smoking ban.

**Methods:**

Experimental measurements of particulate matter 2.5 μm (PM_2.5_), performed during a cross sectional study of 49 hospitality venues and offices in Athens and Crete, Greece during February – March 2006.

**Results:**

Levels of ETS ranged from 19 μg/m^3 ^to 612 μg/m^3^, differing according to the place of measurement. The average exposure in hospitality venues was 268 μg/m^3 ^with ETS levels found to be highest in restaurants with a mean value of 298 μg/m^3 ^followed by bars and cafes with 271 μg/m^3^. ETS levels were 76% lower in venues in which smoking was not observed compared to all other venues (p < 0.001). ETS levels in Greek designated non-smoking areas are similar to those found in the smoking sections of UK hospitality venues while levels in Ireland with a total smoking ban are 89% lower and smoke-free communities in the US are 91 – 96% lower than levels in Greece.

**Conclusion:**

Designated non-smoking areas of hospitality venues in Greece are significantly more polluted with ETS than outdoor air and similar venues in Europe and the United States. The implementation of a total indoor smoking ban in hospitality venues has been shown to have a positive effect on workers and patrons' health. The necessity of such legislation in Greece is thus warranted.

## Background

Passive smoking is a serious threat to public health. It has been shown in numerous studies that prolonged exposure to Environmental Tobacco Smoke (ETS) in adults can predispose them to cardiovascular disease (by damaging the arterial endothelium, and reducing blood high density lipid levels), certain types of cancer, chronic obstructive pulmonary disease and to a dose response relation of lung function impairment [1-7]. Effects of ETS on children are even greater due to their higher metabolism and ventilation rates predisposing them not only to cancer and cardiovascular disease but also to asthma, lower respiratory tract illness, neurological disorders and even impairing cognitive abilities [8-10].

ETS itself comprises sidestream smoke emitted from the smouldering tobacco between puffs and exhaled mainstream smoke from the smoker. Both sidestream and mainstream smoke affects not only tobacco users but also passively other people who share their close environment whether at home, at work or in a car. Over the past years there has been a global movement to ban smoking from public places, initially in places related to health services and then in private sector places, such as offices and hospitality venues. [11].

PM_2.5 _measurement is used to calculate the concentration of particulate matter in the air smaller than 2.5 microns in diameter. Particles of this size are released in significant amounts from burning cigarettes, are easily inhaled deep into the lungs, and are associated with pulmonary and cardiovascular disease and mortality [12]. ETS is not the only source of indoor pollution that can produce PM_2.5 _since particles of this size are not specific to tobacco smoke (ambient particle concentrations arising from cooking or vehicle fumes are also of that size), but PM_2.5 _monitoring is highly sensitive to ETS and high levels of such particles have been attributed almost solely to ETS in hospitality venues [13-16].

Avoiding exposure to ETS in Greece is extremely difficult. Greece has the highest adult smoking prevalence in Europe and one of the highest worldwide. It is estimated that 40% of the adult population are current daily smokers with smoking prevalence differing according to location [17,18]. Currently smoking is forbidden in public service institutions, transport waiting areas and means of transport, health care service centers and educational institutions, but on the other hand permitted in certain areas of hospitality venues and private workplaces. Specifically by law 50% of the indoor area of hospitality venues should be smoke free, and adequate air circulation should be provided for both areas. Taking into account the serious adverse health effects that ETS probably has on the Greek population, the purpose of this study is to quantify the levels of ETS exposure in the designated non-smoking areas in a substantial number of public venues in Greece and to compare them with the air pollution levels found in other venues, nationally and internationally.

## Methods

A TSI SidePak AM510 Personal Aerosol Monitor (TSI, Inc., St. Paul, Minnesota, USA) was used to sample and record the levels of fine particles in the air. The SidePak uses a built-in sampling pump to draw air through the device and the particulate matter in the air scatters the light from a laser to assess the real-time concentration of particles less than 2.5 μm in micrograms per cubic meter, or PM_2.5_. The SidePak's flow rate was set to 1.7 litres per minute to ensure proper operation of the attached 2.5-micron impactor. In accordance with the Global Air Monitoring Study Protocol, a calibration factor of 0.32, which is suitable for tobacco smoke, was applied to all data [19]. Other researchers have confirmed this calibration factor as well [20,21]. In addition, the SidePak was zero-calibrated prior to each use by attaching a HEPA filter according to the manufacturer's specifications so as to determine the zero level of particle exposure. Outdoor levels of PM_2.5 _were measured at an average of 35 μg/m^3^.

### Sampling of venues

A total of 51 venues were sampled in Athens (Athens, Haidari, Kifissia, Nea Erythrea, Penteli, Peristeri) and in Crete (Archanes, Heraklion, Rethymnon) during the months of February and March 2006. The venues were selected to get a broad range of size, location and type of venue that included restaurants, cafés, bars and offices. A convenience sample was used since there is no list of hospitality venues in Crete or Athens from which to choose from. The equipment was set to a one-minute log interval, which averages the previous 60 individual second measurements. Sampling took place during the evening (8 pm-2 am) for hospitality venues since they are mostly visited during those hours, while air measurements from offices were taken during morning, working hours. Sampling was discreet in order not to disturb the occupants' normal behaviour, during which observational information was noted, regarding number of cigarettes, people, air volume and other factors that might affect the data (i.e. candles or cooking in area). The monitor was generally located in a central location on a table or bar of the non-smoking area of the venue (where available although by law at least 50% of the indoor area) and not on the floor, so that the air being sampled was within the non-smoking occupants' normal breathing zone. In all cases air monitoring was performed for at least 30 minutes while the first and last minute of logged data were removed because they are averaged with outdoors and entryway air. The remaining data points were averaged to provide an average PM_2.5 _concentration within the venue. Venues were excluded if there were any apparent sources of particles other than smoking that would act as a confounding factor and would give higher measurements of PM_2.5 _that could not be attributable to ETS. This resulted in the exclusion of one restaurant that had open fire grilling. One other restaurant was also excluded due to incomplete observational data. Analysis of the data was performed at the Roswell Park Cancer Institute.

### Statistical analysis

The primary goal was to record the ETS levels in the venues and then to assess the difference in the average levels of PM_2.5 _in places were smoking was observed and where it wasn't. Additionally, levels of PM_2.5 _in venues where smoking was noticed were compared with levels in venues in the US, the UK and Ireland where there is a comprehensive smoking policy. The data from the US was based on research done in hospitality venues in a number of states [22]. The data from the UK were from smoking areas of pubs (non food serving) and from Ireland only smoke-free pubs [23,24]. All were included as groups for comparison with the data of the present study. Statistical significance is assessed using the Mann-Whitney U-test.

## Results

Smoking in the non-smoking areas was noticed in 46 of the 49 venues sampled. No smoking during measurements was observed only in two offices and one restaurant. Table 1 presents detailed information about each restaurant sampled.

**Table 1:** Descriptive results and PM_2.5 _levels of designated non smoking areas in restaurants in Greece

**Location**	**Volume (m^3^)**	**People (mean)**	**Cigarettes (mean)**	**Smoker Density^1^**	**PM_2.5 _Mean (μg/m^3^)**
Athens	328	36	5.3	1.63	**409**
Athens	304	51	1.7	0.55	**159**
Athens	144	20	2.0	1.39	**129**
Athens	314	42	6.3	2.02	**180**
Athens	631	23	3.3	0.53	**298**
Athens	117	24	2.7	2.27	**420**
Athens	507	10	1.3	0.26	**349**
Athens	150	26	3.7	2.45	**419**
Crete	612	60	8.7	1.42	**322**
Crete	2442	149	20.7	0.85	**541**
Crete	428	60	8.0	1.87	**290**
Crete	92	8	0.0	0.00	**64**
**Mean**	**467**	**39**	**5**	**1.17**	**298**

^1 ^Average number of cigarettes per 100 m^3^

Levels of restaurant indoor air pollution ranged from 64 μg/m^3 ^to 541 μg/m^3^, with a mean value of 298 μg/m^3^. Only one of the restaurants sampled did not have smokers during the time of measurement, and the average level of PM_2.5 _in this venue was 64 μg/m^3^, while in the 11 others in which smoking was evident the mean level of PM_2.5 _was 320 μg/m^3^. The venue smoker density ranged between 0 – 2.45 cigarettes per 100 m^3^, with a mean value of 1.17.

As shown in Table 2, Smoking was observed in all 31 cafes, bars and clubs, with the mean PM_2.5 _levels in cafés and bars ranged between 49 and 612 μg/m^3^, with factors such as venue size, smoker density, open windows, ventilation causing the differences as seen by the observational measurements. The mean PM_2.5 _level of those venues was 271 μg/m^3^.

**Table 2:** Descriptive results and PM_2.5 _levels of designated non smoking areas in Café, Clubs and Bars in Greece

**Type**	**Location**	**Volume (m^3^)**	**People (mean)**	**Cigarettes (mean)**	**Smoker Density^1^**	**PM_2.5 _Mean (μg/m^3^)**
Café^2^	Athens	40	11	2.7	6.63	**221**
Bar	Athens	102	44	14	13.73	**521**
Bar	Athens	93	6	1.0	1.07	**231**
Café	Athens	335	46	14.3	4.28	**372**
Café	Athens	104	36	7.0	6.70	**452**
Café	Athens	150	27	3.7	2.44	**379**
Café	Athens	337	39	7.3	2.17	**214**
Café	Athens	191	17	2.3	1.22	**397**
Café	Athens	208	5	1.0	0.48	**71**
Café	Athens	112	21	5.0	4.47	**133**
Café	Athens	59	16	5.0	8.48	**175**
Café	Crete	125	25	5.0	3.99	**156**
Café	Crete	847	118	19	2.24	**332**
Café	Crete	159	20	5.3	3.36	**442**
Café	Crete	216	7	0.3	0.15	**49**
Café	Crete	190	35	9.0	4.73	**612**
Café	Crete	160	14	2.7	1.67	**108**
Café	Crete	520	25	4.7	0.90	**66**
Café	Crete	411	62	11.0	2.68	**339**
Café	Crete	318	51	11.7	3.67	**171**
Café	Crete	183	42	5.7	3.09	**173**
Café	Crete	153	10	1.3	0.87	**281**
Café	Crete	85	14	2.7	3.14	**412**
Café	Crete	1657	40	9.7	0.58	**121**
Café	Crete	230	20	7.3	3.18	**322**
Café	Crete	219	22	4.3	1.97	**143**
Café/Bar	Athens	391	27	5.7	1.45	**265**
Café/Bar	Athens	734	29	4.3	0.59	**97**
Café/Bar	Crete	411	34	8.0	1.95	**322**
Café/Bar	Crete	196	47	10.7	5.44	**520**
Café/Bar	Crete	883	53	17.0	1.93	**296**
**Mean**		**317**	**31**	**6.7**	**3.2**	**271**

^1 ^Average number of cigarettes per 100 m^3^

^2 ^Café located in a private hospital

Table 3 presents the measurements taken in offices and banks. Smoking was not observed in only two of the offices sampled, in the three other offices and one bank, smoking was observed, although prohibited. In the offices where smoking was not observed during measurements the PM_2.5 _levels ranged between 39 and 63 μg/m^3 ^and in those where smoking was observed during measurements PM_2.5 _levels ranged between 19 and 236 μg/m^3^. Overall the mean PM_2.5 _level was 88 μg/m^3^, with 51 μg/m^3 ^and 107 μg/m^3 ^found in offices were smoking was not observed and observed, respectively.

**Table 3:** Descriptive results and PM_2.5 _levels of designated non-smoking areas of offices and banks in Greece

**Type**	**Place**	**Volume (m^3^)**	**People (mean)**	**Cigarettes (mean)**	**Smoker Density^1^**	**PM_2.5 _Mean (μg/m^3^)**
Office	Athens	283	9	2.7	0.94	**118**
Office	Athens	59	3	0.3	0.57	**19**
Bank	Crete	4672	82	1	0.02	**53**
Office	Athens	30	2	0	0	**63**
Office	Athens	4	3	0	0	**39**
Office	Athens	4	2	0,7	16.75	**236**
**Mean**		**842**	**17**	**0.8**	**3**	**88**

^1 ^Average number of cigarettes per 100 m^3^

**Figure 1:**
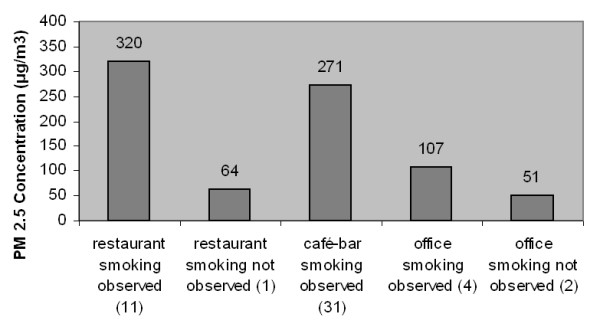
Average levels of ETS in Greece, according to venue area and smoking status measured in PM_2.5 _μg/m^3^.

**Figure 2:**
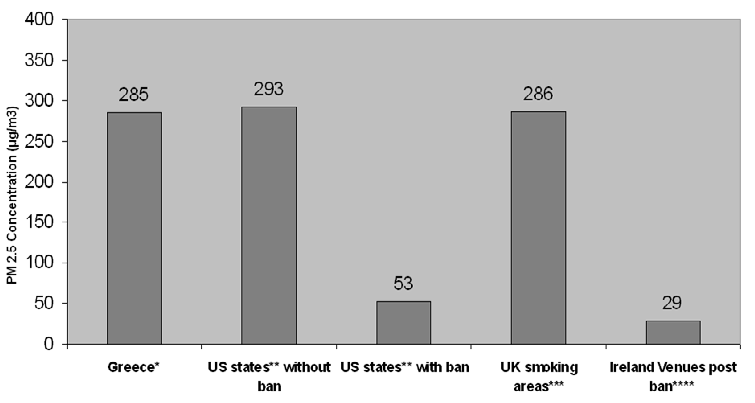
Average levels of ETS in restaurants, cafes and bars in Greece compared to the US, the UK and Ireland before and after the implementation of smoking bans measured in PM_2.5_in μg/m^3^. *Restaurants, cafes and bars in Greece (combined from tables 1 and 2 in non-smoking areas) **States measured before and after a smoking ban [18] *** Smoking areas [23] **** Ireland [24].

Averaged across each type of venue, the lowest levels of indoor air pollution were found in offices and banks (88 μg/m^3^) and the highest levels were found in restaurants (298 μg/m^3^). Café's and bars were found to have PM_2.5 _levels between those two levels (271 μg/m^3^) (Figure 1). In general, the level of indoor air pollution was 76% lower in venues where smoking was not noticed compared to venues where smoking was observed (13 restaurants and 31 cafes and bars), and this difference was statistically significant (p < 0.001). Figure 2 compares the levels of PM_2.5 _before and after the implementation of a smoking ban in the US, the UK, and Ireland to the levels found in venues in Greece.

The observational data in Tables 1, 2 can be combined with the measured levels of ETS so as to model the air exchange rate in each of the measured venues. Assuming a background level of PM_2.5 _= 35 μg/m^3^, the PM_2.5 _levels due to ETS can be calculated for restaurants at 263 μg/m^3 ^(298 μg/m^3 ^- 35 μg/m^3^) and for bars and cafes at 236 μg/m^3 ^(271 μg/m^3 ^- 35 μg/m^3^). Combining this data with the number of active smokers per 100 m^3 ^and using the model developed by Repace et al [13] it can be estimated that restaurants were lower ventilated (2.89 air changes/hour) when compared to bars and cafes (8.7 air changes/hour). The similar levels in PM_2.5 _between both can be attributed to the higher smoker density in cafes and bars compared to restaurants (3.2 vs 1.17)

## Discussion

Venues in Greece are heavily polluted by ETS, with a substantial difference in PM_2.5 _levels between areas were smoking was noticed and areas where it was not noticed, although not prohibited (76% difference). To our knowledge, concentrations of nicotine as a marker of ETS exposure has been measured once before in Greece but only in a few venues [25]. We note that since 2002 there is a law (Health Law 76017) that states that 50% of interior space of bars, cafés, and entertainment centres must be reserved for non-smokers, separated physically if possible, with adequate signs and air circulation [26]. The present study clearly demonstrates that this law (Health Law 76017) is ineffective since the PM_2.5 _levels were found to be elevated in almost all venues sampled even though the measurements were taken in non-smoking areas. Loopholes in this law do exist since bars, cafes and restaurants in Greece use outdoor areas (many of which are covered and walled) most of the year taking advantage of the mild Mediterranean climate [27,28]. The enforcement of the legislation is very difficult since the ban is only applicable to indoor areas, thus excluding many venues that require only a small indoor area for their clientele in winter. The effect of air circulation becomes obvious when comparing the levels of smoker density, ETS and air circulation between the different venues of this study. Cafes and bars are better ventilated than restaurants, due to the fact that they are usually equipped with better ventilation systems since after a certain time of the night their doors must be closed so as to reduce noise pollution, while restaurants (due to lower levels of noise pollution) are not obliged to do so.

In the United States, the EPA cited over 80 epidemiological studies to create a particulate air pollution standard in 1997 [29]. In order to protect public health, the EPA has set limits of 15 μg/m^3 ^as the average annual level of PM_2.5 _exposure. Based on the latest scientific evidence, the EPA currently proposes even lower PM_2.5 _standards to adequately protect public health [12,30]. This further highlights the concern of high PM_2.5 _exposure of people in smoking environments.

Previous studies have evaluated air quality by measuring the change in levels of PM_2.5 _between smoke-free venues and those that permit smoking. Repace studied 8 hospitality venues in Delaware before and after a state-wide prohibition of smoking in these types of venues and found that about 90% of the fine particle pollution could be attributed to tobacco smoke [13]. Similarly, in a study of 22 hospitality venues in Western New York, Travers et al., found a 90% reduction in PM_2.5 _levels in bars and restaurants, and an 84% reduction in large recreation venues such as bingo halls and bowling alleys after the smoking ban's implementation [16]. Another cross-sectional study of 53 hospitality venues in 7 major cities across the U.S. showed 82% less indoor air pollution in the locations subject to smoke-free air laws, even though compliance with the laws was less than 100% [22]. Compared to Greece, ETS levels in Ireland (with a full ban) are 89% lower, while in the UK findings are rather similar to those in Greece since, average ETS exposure was estimated at 277 μg/m^3 ^in non-food serving pubs. In this study, Edwards et al also noted differences in ETS exposure depending on the deprivation of the surrounding area, with deprived areas found to have higher levels of exposure compared to more affluent areas (366 μg/m^3 ^vs 187 μg/m^3^. [23,24]. In New York, Delaware, and Laramie Wyoming, which have comprehensive smoke-free air legislation, ETS levels are currently 91%, 92%, and 96%, respectively, lower than levels in hospitality venues in Greece [13,31,32].

Other studies have directly assessed the role ETS exposure on human health. One study found that respiratory health improved rapidly in a sample of bartenders after a state smoke-free workplace law was implemented in California (and another study reported a 40% reduction in acute myocardial infarctions in patients admitted to a regional hospital during the 6 months that a local smoke-free ordinance was in effect [33,34]. Farrelly et al. 2005, also showed a significant decrease in both salivary cotinine concentrations and sensory symptoms in hospitality workers after New York State's smoke-free law prohibited smoking in their worksites [35].

### Study limitations

In the present study, the venues sampled were not selected randomly from a list of all venues in Crete and Athens, since no such list exists. Therefore we cannot conclude that these levels are representative of the levels found in other venues in Greece. Also measurements were taken per venue during one night of the months February and March, during which the venues have their windows closed, while we were unable to have information on the exact ventilation rates (although they were calculated by combining observational and experimental measurements). During summer months, most venues have panel windows that slide open and therefore it is possible that the levels of PM_2.5 _during the summer months could be lower than those measured in spring. Biomarker evaluation was not performed to asses ETS exposure in this study due to the fact that cotinine, the major metabolite of nicotine in the body has a half life of 19 hours and therefore would not depict the exposure in those venues but the participants collective ETS exposure of the past day. Despite the above limitations, this is the first time that PM_2.5_levels, as a marker of ETS exposure, were measured in Greece and compared with measurements taken in the US, UK and Ireland before and after the implementation of a total smoking ban in hospitality venues.

## Conclusion

Hospitality venues allowing indoor air smoking in Greece are significantly more polluted than indoor smoke-free sites or outdoor air in Greece, and venues in the US, UK and Ireland. The present study demonstrates that even in the designated non smoking areas, workers and patrons are exposed to harmful levels of ETS, a known human carcinogen and toxin. The implementation of a total smoking ban in such hospitality venues in Greece would have a significant positive effect on the population's health, if it were enforced effectively. Unfortunately, since the Greek population adheres to the classical Mediterranean libertarian ideas of freewill and choice of lifestyle, there is an inherent loath to comply with any laws that restrict personal freedom, making the enforcement of such laws difficult. There is also a lack of systematic health education campaigns regarding smoking that would possibly nurture a smooth transition between old and new smoking policies in Greece, and hardly any existing mechanisms that would supervise their implementation.

Even so, laws that prohibit smoking in public work places, such as those enforced and adhered to in the US, UK and Ireland, would dramatically reduce second-hand smoke exposure and improve worker and patron health if enforced in Greece.

## Competing interests

The author(s) declare that they have no competing interests.

## Authors' contributions

CIV collected data, participated in the study design and was the main author of the manuscript. BK collected data, participated in its design and helped to draft the manuscript. MJT provided training in data collection, analyzed the data, and helped draft and edit the manuscript. EP and YT collected data and helped to draft the manuscript. AK participated in its design and helped to draft the manuscript. All authors read and approved the final manuscript.

## Pre-publication history

The pre-publication history for this paper can be accessed here:


